# Intraoperative cell savage for life-threatening haemorrhagic caesarean section

**DOI:** 10.1186/cc12300

**Published:** 2013-03-19

**Authors:** AS Ducloy, C Barre-Drouard, E Jaillette, S Depret-Mosser, B Wibaut, B Vallet, S Susen

**Affiliations:** 1 Hôpital Jeanne de Flandre, Lille, France; 2 Centre hospitalier regional universitaire Lille, France

## Introduction

Postpartum hemorrhage (PPH) remains the leading cause of maternal mortality. After IOCS introduction in our north of France tertiary-care obstetric unit, the consecutive cohort of severe PPH was collected prospectively and compared regarding the use of IOCS or not.

## Methods

A case-control prospective open-label study comparing the management and outcome of a consecutive cohort of 324 severe PPHs in elective and emergency hemorrhagic caesarean sections (CS) over 3 years.

## Results

IOCS was used in 70 severe PPHs and 254 severe PPH controls were managed without IOCS. Placenta accreta can be selected as the best indication for RBC restitution. In the 1,500 to 3,000 ml PPH, allogeneic transfusion was decreased in the IOCS group: 17.6 versus 56.3% (*P *= 0.006); PRBC: 0 (0 to 3) versus 3 (0 to 6) (*P *= 0.045). IOCS spared 87 blood bank PRBC (17,374 ml); that is, 24.2% of the total transfusion need. No amniotic fluid embolism has been observed in the group with IOCS whereas one case appeared in the control group without IOCS.

## Conclusion

Regarding the literature [1-4] and our study, IOCS could be used safely in PPH during CS. A leukocyte filter for retransfusion has been recommended and Rhesus isoimmunization must be precluded and monitored by repeated fetal RBC testing.
